# Enhancement drugs: are there limits to what we should enhance and why?

**DOI:** 10.1186/1741-7015-8-50

**Published:** 2010-08-03

**Authors:** Morten Hesse

**Affiliations:** 1 University of Aarhus, Centre for Alcohol and Drug Research, Artillerivej 90, 2nd 2300 Copenhagen S, Denmark

## Abstract

Substances, such as alcohol, opiates and cannabis, have been used by humans for millennia. Today, a much wider range of substances are used for a range of purposes, including the enhancement of performance during university studies, sexual experiences, sports, exercise, at celebrations, socializing and the experience of art and music. Substance use is also associated with a range of harmful effects to the individual and society as a whole. Prohibitions, regulation, prevention and treatment have all been used to protect against this harm. In this commentary, it is argued that public health interventions should target relevant harms and not to evaluate which aspects of human endeavors and experiences should be enhanced and which should not. It is argued that interventions should directly target the harmful effects, using the best available evidence. Two examples are given of substances that may be altered to prevent serious harm - one for alcohol and one for cannabis. In the case of alcohol, the addition of dissolved oxygen could reduce both the risk of accidents and the risk of liver damage associated with alcohol consumption. In the case of cannabis, there is strong indication that the reduction of content Δ-tetrahydrocannabinol and the increase of cannabidiol could reduce the risk of psychoses and the addiction associated with its use. The aim of this article is to show that responsible regulation should not necessarily be restricted to preventing the use and/or (in the case of alcohol) a reduction in the amounts and frequency of its use, but should also aim to include a range of other strategies that could reduce the burden of illness associated with illicit substance use.

## Background

Psychoactive substances such as alcohol, cannabis and opiates have been used by humans for thousands of years and, in recent times, a multitude of new substances have been added to the list of such substances. The reasons for the use of such substance are diverse. They range from alleviating pain and hunger, obtaining contact with a sacred world and intoxication.

In ancient Greek mythology, the god of wine, Dionysus, is also known as the liberator [1]. The god was believed to liberate people from their normal self by 'madness, ecstasy or wine'. Over 5000 years ago, the Sumerians in Mesopotamia called the poppy the 'joy plant' [2]. In recent decades, a number of drugs have been developed to treat mental disorders. A range of drugs have also been used to obtain other effects: Sildefanil is used by men who do not have erectile dysfunction [3] and anabolic steroids are being used to increase the size of muscles [4].

While such substances have been used by humans for many years, societies have also observed that substance use leads to problems. In recent centuries, these concerns have lead to national and international legislation which regulates the production, manufacturing and use of these substances.

## Discussion

### Broadening the scope

Currently, substances are being manufactured and altered in ways that lead to many hazards for those who use them.

A recent editorial in Nature suggested that one type of use of substances - the use of drugs to enhance academic work - should be accepted as a fact and regulated responsibly, rather than simply rejected as cheating or dangerous behaviour [5]. II suggest that this view may be broadened beyond regulating substance use for cognitive enhancement. As drugs are not only used to enhance cognitive functioning, the responsible use of substances may extend beyond these purposes. The point is that public health researchers, policy makers and legislators should take responsibility for regulation of use- not simply reject or accept a particular substance.

The heart of the problem is the definition of the aims of these policy makers and legislators. Is the goal of policy to maintain public order and protect the public from harm, or is it to define what the accepted view of what a good life is and how the good citizen should behave?

It is important to discriminate between the prevention of harm and the definition of a good life and the good citizen. Although it is not without pitfalls, the prevention of harm is a legitimate goal of policy and public health. However, imposing a view of what the good life should be is more questionable. Public health concerns can be good reasons to prohibit or restrict the manufacturing and sale of goods that could be harmful. On the other hand, public health researchers and policy makers should not judge what in life should be enhanced. Whether students or other people wish to have their cognitive skills enhanced or if they wish to obtain a state that can be characterized as ecstatic or even 'mad' is not something that should be the concern of health authorities.

In a modern society, using drugs for the enhancement of the experience of music, dancing, sex or conversation at a dinner party, should be no more or less a legitimate goal of an individual than the enhancement of the ability to study for an exam.

Substances that affect the body vary in terms of their harm, including risk of addiction [6], and use of substances can lead to a variety of adverse health effects and may negatively affect society [7]. Harm may vary between substances and both experts and drug users generally perceive these harms differently from the legislators [8]. It our belief that it should be the harmful effects of drugs of addiction that should guide decisions about the regulation of substances rather than the reason for their use. Indeed, the reduction of such harm should be the very target of policy and research, regardless of the intentions of the drug user.

### Some examples: changing the contents of substances used for non--medical purposes

In the following, I shall describe how substances that are already being used for non-medical purposes may be changed in ways that can potentially reduce their negative impacts on individuals and on society as a whole. The point is that regulation should not target why and how people use substances but only how the harm associated with substance use can be reduced or avoided. This includes the risk of addiction: since the risk of addiction varies from drug to drug, it follows that the ingredients could be altered in ways that increase or decrease the risk [6].

One substance that can possibly be altered in order to reduce some of its negative side-effects is alcohol. Alcohol is one of the most commonly used intoxicating substances and contributes substantially to the burden of illness worldwide. It is estimated that the health risk associated with alcohol is five times greater than the burden associated with that of the use of all illicit drugs combined [9]. In a Korean study, dissolved oxygen was added to alcoholic beverages [10]. The effect was a much faster elimination of alcohol in the blood. Potentially, the faster elimination could lead to a much quicker restoration of normal functioning in social drinkers, thus reducing the risk of accidents and other side effects of alcohol drinking. The addition of oxygen could also potentially reduce the risk of liver damage and acute hangover symptoms. The real-life significance of this finding is unknown and requires further study. For instance: do alcohol drinkers increase their consumption over a period of drinking in order to maintain the same blood-alcohol concentration, offsetting the potential benefits of a manipulated alcohol product?; and what is the impact of the addition of dissolved oxygen on the risk of developing an addiction?

Another substance that is commonly used in countries such as the US, southwest Europe and Australia, is cannabis [11]. Cannabis is associated with: some risk of accidents, although less so than alcohol; low birth-weight of the fetus when smoked during pregnancy; and some increased risk of diminished cognitive abilities [11]. There is also considerable evidence that cannabis use produces sub-clinical psychotic symptoms in regular users, especially when the age of onset is below 15 [12], and that cannabis use is a risk factor for subsequent schizophrenia [13]. While the real-life importance of the link between cannabis use and psychosis is disputed [11], the evidence overall points to a dose-dependent association between cannabis use and the development of schizophrenia spectrum disorders. However, if cannabis products were regulated, the content of cannabis products could potentially be changed, so that the risk of psychosis could be reduced [14].

Cannabis contains two important substances that appear to have almost opposite effects, namely Δ-tetrahydrocannabinol (THC) and cannabidiol. In an study of psychotic symptoms among cannabis users, Morgan and Curran showed that in hair analyses the use of THC without cannabidiol was strongly correlated to elevated scores on a scale representing psychosis proneness; when cannabidiol was also present, the scores became similar to those of non-cannabis using controls [15]. If cannabis was regulated rather than prohibited, it would be possible to regulate both the amount of cannabidiol and THC in cannabis products to reduce the risk of addiction and psychosis, by balancing the amount of cannabidiol and THC. The current state of affairs is that drugs are being bred to contain increasing amounts of THC [16]. An increase in the amount of THC used in cannabis may have contributed to an increase in the disorders which cause cannabis users to seek medical treatment in the US and many European countries [11] and the use of these cannabis products is particularly common among patients who present with a psychotic disorder [17]. In this way, prohibition may push cannabis in the wrong direction, making a harmful drug worse. The regulation of this drug and its ingredients could make it less harmful.

### The implications

The goal of a drug policy (for lack of a better term) should be the responsible regulation of the range of substances that have the potential to influence psychological and physiological processes. Research can help not only to identify potential harm and to develop effective strategies to regulate substances but also to alter substances or develop new chemical properties which could result in fewer negative consequences.

Essentially, changes in the content of substances can be thought of as a parallel to regulating motor vehicles. Such regulation should not address where people should drive, or why. However, regulation can make it mandatory for cars to have seat belts, rearview mirrors and other equipment that reduces the risk of accidents.

Changing the harms associated with substances should obviously not be restricted to changing the chemical properties of substances. Equally important is the development of strategies to control access to substances, where harm remains a risk. Also, regulating how drugs can be consumed may sometimes be a feasible strategy. For instance, if cannabis users can be helped to abandon smoking and use so-called vaporizers, they will reduce the amount of carbon-monoxide that they consume along with cannabis, dramatically reducing the harm associated with cannabis [18].

Regulating traffic does not eliminate the risks associated with traffic. Accidents will always happen and the driver's behaviour will still be an immensely important factor in the risk of accidents. In all likelihood, some risk will remain with substances that alter the functioning of somatic processes, whether they are primarily located in the brain or primarily located elsewhere. This risk will vary substantially between users, depending on their behaviour.

The regulation of the chemical content of substances that influence bodily processes drugs and the regulation of price and accessibility should be handled intelligently and effectively and should not be based on a moral perspective. The intelligent regulation may at times be a complete prohibition or a restriction that leads to its being used for medical purposes only. At other times it may regulate the contents of substances or where and at what price they can be sold. Any course of action will produce costs but they may also produce benefits. Again, the important thing is to always define which is the more, and which is the less, harmful course of action based on the best available evidence.

## Conclusion

Substances have been used for millenniums to enhance experiences and performance. Substance use may also cause harm, such as accidents, illness and addiction, and yet some people still choose to use them. The task of public health interventions should be to target the health-related harms, but not to evaluate which aspects of human endeavors and experiences should or should not be enhanced. Responsible regulation would not necessarily be restricted to preventing the use of a potentially harmful substance but may include a range of other strategies that can reduce the burden of illness associated with substance use. One example given in this commentary is the changing of the chemical content of substances in order to reduce certain risks associated with a given substance.

## Abbreviations

THC: Δ-tetrahydrocannabinol

## Competing interests

The author declares that they have no competing interests.

## Authors' contributions

MH wrote the manuscript.

## Pre-publication history

The pre-publication history for this paper can be accessed here:

http://www.biomedcentral.com/1741-7015/8/50/prepub
